# Differences in sexual risk behaviours, HIV care utilisation and experiences of stigma between transgender women and cisgender men who have sex with men: findings from integrated biobehavioural surveys in Ukraine 2013–2018

**DOI:** 10.1136/bmjopen-2025-104918

**Published:** 2026-02-10

**Authors:** Saher Aijaz, Peter Vickerman, Tetiana Saliuk, Jane Nicholls, David Gillespie, Kerenza Hood, Jack Stone

**Affiliations:** 1 Bristol Medical School, University of Bristol, Bristol, UK; 2 Population Health Sciences, University of Bristol, Bristol, UK; 3 International Charitable Foundation “Alliance for Public Health”, Kiev, Ukraine; 4 Cardiff University, Cardiff, UK; 5 College of Biomedical & Life Sciences, Cardiff University Centre for Trials Research, Cardiff, UK; 6 Population Health Sciences, University of Bristol Medical School, Bristol, UK

**Keywords:** Transgender Persons, Sexual and Gender Minorities, HIV & AIDS

## Abstract

**Abstract:**

**Objectives:**

To assess whether transgender women who have sex with men (TGWSM) sampled in men who have sex with men (MSM) biobehavioural surveys in Ukraine experience different levels of sexual risk, stigma, HIV prevalence and engagement in the HIV care than cisgender MSM (CMSM).

**Design:**

Analysis of secondary data from three population-level cross-sectional surveys.

**Setting:**

The analysis was conducted on data from three rounds of integrated biobehavioural surveys of MSM in 27 cities of Ukraine from 2013 to 2018.

**Participants:**

Data from n=18 621 MSM with n=18 102 CMSM and n=503 TGWSM.

**Primary and secondary outcome measures:**

The primary outcomes were differences in sexual risk behaviours, HIV testing and treatment uptake, and the secondary outcomes were differences in lifetime experiences of stigma, coercive sex and physical assault (in the 2018 survey only) between CMSM and TGWSM.

**Results:**

Compared with CMSM, TGWSM were more likely to be clients of non-governmental organisations (adjusted OR, aOR: 1.39, 95% CI 1.15 to 1.67), engage in commercial sex (last month; aOR: 1.28, 95% CI 1.01 to 1.61), have group sex (aOR: 1.31, 95% CI 1.06 to 1.61), more long-term sex partners (last month; adjusted incidence rate ratio: 1.14, 95% CI 1.03 to 1.27), history of imprisonment (aOR: 1.51, 95% CI 1.00 to 2.31) and engage in chemsex (last month, aOR: 1.58, 95% CI 1.12 to 2.23). We found no difference in HIV prevalence (5.17% in TGWSM vs 5.43% in CMSM, p=0.065). In 2018, more TGWSM reported lifetime experience of stigma from family and friends (aOR: 3.58, 95% CI 2.54 to 5.04), general social stigma (aOR: 3.13, 95% CI 2.22 to 4.41), anticipated healthcare stigma (aOR: 3.63, 95% CI 2.53 to 5.16), physical assault (aOR: 2.73, 95% CI 1.85 to 4.03) and coercive sex (aOR: 3.01, 95% CI 1.99 to 4.55) than CMSM.

**Conclusions:**

TGWSM in Ukraine may be at increased risk of HIV acquisition compared to CMSM due to many factors including elevated levels of stigma and violence. Services specifically tailored for transgender people are needed to help reduce these high-risk behaviours.

**STRENGTHS AND LIMITATIONS OF THIS STUDY**

- We used a large nationally representative dataset to study the HIV risk differences between transgender women having sex with men and cisgender men having sex with men.
- Possible misclassification of participants as transgender women.
- The stigma instrument may not have captured transgender-identity specific stigma and so is likely to underestimate levels of stigma experienced by transgender women who have sex with men.
- Potential for recall bias and social desirability bias.
- Under-representation of older individuals may limit generalisability.

## Introduction

 Eastern Europe and Central Asia (EECA) remains the epicentre of the fastest growing HIV epidemic in the world, with 160 000 new HIV infections in 2021.^1^ HIV infections have increased by 48% since 2010 and there is suboptimal coverage of HIV treatment, with only half of all people living with HIV accessing antiretroviral therapy (ART) in 2021.^2^ As of 2020, the incidence of HIV in Ukraine was the second highest in the region.^1^

In EECA, as per UNAIDS, key populations, including men who have sex with men (MSM), transgender people, sex workers, people who inject drugs (PWID), and people in prisons and other closed settings, have a high burden of HIV, with HIV prevalence in 2022 estimated as 3.9% among MSM, 20.9% among PWID and 3.1% among sex workers in Ukraine.^3^ Less is known about the burden of HIV in transgender populations in EECA. In 2020, data suggested that HIV prevalence among transgender people was 1.7%, lower than the prevalence among MSM (3.9%) but higher than general population prevalence (0.9% in 2021 for 15–49 years).^4 5^

Evidence suggests that stigma and discrimination based on the gender identity of transgender persons can limit their engagement with HIV treatment and prevention services,^6 7^ while there is inadequate access to HIV services that are tailored to the needs of transgender persons globally.^8^ HIV transmission in this group is marked by entanglement of multilevel biopsychosocial factors,^9^ however, social, structural and behavioural contexts play a profound role in shaping the HIV-related vulnerabilities in these populations. At the individual level, behaviours such as engagement in high-risk sexual activities and substance use increase HIV acquisition risk.^9^,^11^ These are often entangled with structural and societal level factors like stigma, discrimination and lack of legal protection and recognition of the rights of transgender individuals, which further compromises the mental health of these individuals.^7 9 12 13^ Additionally, these multilevel factors cause inequalities in education, health access and employment. All of these factors sustain HIV vulnerabilities by creating a syndemic environment compounding HIV risk in such populations.^9^

Research has also indicated lower levels of health-seeking by transgender persons.^14^ While there is a lack of comprehensive research exploring the underlying causes, some evidence attributed this to stigma and discrimination faced at various levels: structural, societal and individual.^14^ Evidence has also indicated that internalisation of discriminatory attitudes and prejudice by transgender persons underlies these low levels of healthcare utilisation.^14^ This stigma can also lead to reduced knowledge about HIV prevention and condom usage.^15^ A strong body of research has highlighted negative associations between structural and individual-level stigma and utilisation of HIV care, HIV testing and Pre-Exposure Prophylaxis (PrEP) use among diverse population groups.^16^,^19^ Research also established links between stigma and reduced sexual health services utilisation due to and perceived fear of stigmatisation by healthcare professionals among sexual and gender-minority populations.^17 19^ The intersection of homophobic and transphobic stigma with HIV status, sex work, ethnicity and drug use is also believed to underlie adverse HIV outcomes among populations.^20 21^

While evidence suggests a higher risk for HIV acquisition among key populations,^22^ limited research is available from the EECA about the influence of these factors on the HIV treatment and prevention cascade among MSM and transgender populations.^23^ There are also limited data from EECA on levels of sexual behaviours, engagement with HIV care cascade, HIV prevalence and levels of stigma experienced by transgender persons, and how that compares to other population groups. In this study, we analysed three national integrated biobehavioural surveys (IBBS) of MSM in Ukraine that included transgender women who have sex with men (TGWSM), to assess the differences in these indicators between TGWSM and cisgender MSM (CMSM).

## Methods

### Data characteristics

This analysis uses data collated from three national IBBSs for MSM in Ukraine covering the years 2013, 2015 and 2018. Briefly, IBBSs are used to monitor HIV epidemics by combining biological testing for HIV and other infections with surveys of behaviours and knowledge. Data from earlier surveys were not used as they did not include questions to stratify participants according to transgender status. Individuals who were presumed assigned male at birth, who self-reported sexual contact (oral or anal) with a man in the previous 6 months, were over 14 years of age, and were resident, employed or studying in one of the cities where the survey was conducted, were eligible to participate. This eligibility criteria allowed for the inclusion of transgender people who were otherwise described as MSM in the survey and defined to be born as ‘biologically men’ and practise sex with men in the past 6 months^24^; transgender status was assessed from a question related to transgender identity. Participants were recruited using respondent-driven sampling (RDS), full details of which can be found in IBBS 2013,^24^ 2015^25^ and 2018^26^ reports. The surveys were conducted in 28 cities of Ukraine.

Using a standardised questionnaire, the IBBS surveys collected self-reported data on the demographic and behavioural characteristics of participants, including sexual risk behaviours, health-seeking practices, HIV testing history, HIV treatment uptake and knowledge of HIV transmission. Dried blood specimens were collected for HIV testing.

#### Gender identity and sexual behaviour variable

Transgender status in the IBBS survey was assessed using a binary response question. The inclusion criteria for the overall study population were if the sex assigned at birth was male and the individual had had sex with other men in the past 6 months with other men. Accordingly, in this study, we refer to this subgroup as transgender women having sex with men, while some individuals in the transgender group in IBBS may include inter alia individuals including transgender people and those who identify with non-binary or other gender identities. Details of the transgender item and definition of MSM, as used in the survey, are given in online supplemental table 1.

### Statistical analyses

Data from the three surveys were pooled and analysed using Stata/MP vV.16.1. Transgender status was characterised using responses to the question ‘Do you consider yourself as transgender’. Differences in sexual behaviours and healthcare seeking behaviours between CMSM and TGWSM were assessed by χ^2^ test or t-test for categorical and continuous variables, respectively. Using mixed-effects logistic regression with city and year as crossed random effects, we assessed associations between transgender status and sexual risk behaviours (provision of commercial sex in the last year, procurement of commercial sex in the last month, engaging in group sex with both men and women in the last 30 days, engaging in group sex with men in the last 30 days, engagement in chemsex (sex under the influence of recreational drugs) in the last 30 days, HIV positivity, lifetime Experience of imprisonment and access to services (being a client of a non-governmental organisation (NGO)—that is, programmes providing HIV prevention, testing and linkage to care services,^27^ receiving free condoms in the last 30 days and, among those testing HIV positive, self-reporting being registered at an AIDS centre and/or being on ART). To account for potential confounding, we adjusted for age (as a continuous variable) and higher education attainment (completed undergraduate or higher degrees) by including these as additional covariates in our models. Age was selected since the vulnerability to HIV and engagement with HIV care could vary across the life course. Education attainment was selected to account for the potential influence of sociodemographic position on HIV outcomes and gender affirmation. Associations between transgender status and number of long-term or casual sex partners were assessed using mixed-effects negative binomial regression models. We did not apply RDS weights in our analyses as its application in regression analysis remains contested due to their negative effects on variance and model stability as RDS weighting may result in increases in variance which can limit the validity of regression models.^28 29^

Analyses considering differences in experience of stigma and violence were conducted using data from the 2018 survey only as these variables were not available in previous surveys. Stigma and violence measures included any lifetime experiences of the following: stigma from family and friends; general social stigma; anticipated healthcare stigma; physical assault; coercive sex; and fear of being in public. These measures were derived from a 15-item inner homophobia scale (online supplemental tables 5 and 6). Differences in experiences of stigma between TGWSM and CMSM were compared using χ^2^ test. We used multivariate logistic regression to assess associations between each stigma and violence outcome and transgender status, age, history of imprisonment, higher education and HIV status. Average marginal effects using the marginaleffects package in R were also measured to quantify the effect of TGWSM and CMSM statuses on the probability of the outcome for models that established statistically significant associations in adjusted analyses. To compare the predicted probabilities between the two groups, we used the *emmeans* package in R.

### Patient and public involvement

Patients and/or the public were not involved in the design, or conduct, or reporting, or dissemination plans of this research.

## Results

### Comparing TGWSM and CMSM

Data from n=18 621 participants (n=8100 from 2013, n=4550 from 2015 and n=5971 from 2018) were analysed to measure differences in HIV prevalence and sexual risk behaviours between TGWSM and CMSM. Of these 18 621 participants, 2.7% (n=503) identified as transgender. Table 1 represents characteristics of the whole sample and how they differ by TGWSM and CMSM status. The mean age of the participants was 28.8 (SD 9.04) years, with those identifying as TGWSM being younger than CMSM (27.67 (SD 8.78) compared with 28.83 (SD 9.04) years (p=0.004). Overall, 2.08% of participants had a history of imprisonment, with more TGWSM having been previously imprisoned than CMSM (3.26% vs 2.05%, p=0.05). A third (12.6%) of participants had an undergraduate or higher degree, with more CMSM (33.3%) having attained higher education than TGWSM (22.2%, p<0.01). A higher proportion (5.43%), although not statistically significant (p=0.49), of CMSM were HIV positive than TGWSM (5.17%, p=0.06).

**Table 1:** Demographic characteristics, sexual practices and behaviours, linkage with HIV service utilisation, and negative experiences overall and for TGWSM and CMSM

	Variable	Total		CMSM		TGWSM		**P value**
		**n=18 621**	**(%)**	**n=18 102**	**97.30%**	**n=503**	**2.70%**	
Demographic characteristics	Mean age (years)	28.8 (SD: 9.04)		28.83% (SD: 9.04)		27.67 (SD 8.78)		0.004
	History of imprisonment	260	2.08%	249	2.05%	11	3.26%	0.052
	Higher education	4104	12.60%	4031	33.31%	73	22.19%	<0.01
Sexual risk behaviours	Provided commercial sex (lifetime)	2835	15.24%	2742	15.17%	93	18.49%	0.004
	Procured commercial sex (30 days)	757	4.07%	732	4.05%	25	4.98%	0.296
	Group sex (6 months)*	2502	20.54%	2409	19.90%	93	27.60%	0.013
	Group sex with men only (6 months)*	2429	19.52%	2340	19.33%	89	26.41%	0.001
	Mean number of long-term anal sex partners (30 days)	0.78 (SD: 1.14)	–	0.7 (SD: 1.13)	–	0.8 (SD: 1.32)	–	0.04
	Mean number of casual anal sex partners (30 days)	1.51 (SD: 2.62)	–	1.5 (SD: 2.60)	–	1.69 (SD: 3.26)	–	0.112
	Mean number of partners they paid for anal sex (30 days)	0.05 (SD: 0.40)	–	0.05 (SD: 0.40)	–	0.08 (SD: 0.39)	–	0.056
	Chemsex in last 30 days†	889	4.78%	850	4.70%	39	7.77%	0.001
	Bought condoms (30 days)	11 989	64.39%	11 645	64.34%	334	66.40%	0.341
	Used condom for last anal sex (recent)*	14 107	75.76%	13 722	75.80%	373	74.16%	0.395
	Always used condom for anal sex (30 days)*	11 886	63.83%	11 575	63.94%	303	60.24%	0.088
	Received free condoms (12 months)	9127	49.01%	8826	48.76%	295	58.65%	<0.001
Engagement with HIV care	Ever tested for HIV	12 642	77.23%	12 294	77.22%	340	77.98%	0.71
	Tested for HIV last 12 months	14 291	76.75%	13 907	76.83%	371	73.75%	0.108
	HIV positive status (self-disclosed)	331	1.78%	325	1.80%	6	1.19%	1.01
	Tested Positive for HIV	1009	5.42%	983	5.43%	26	5.17%	0.065
	Registered at an AIDS centre‡	294	90.18%	291	90.37%	3	75.00%	0.34§
	On antiretroviral therapy¶	209	71.09%	207	71.13%	2	66.67%	1§
	Client of an NGO	6036	36.01%	5841	32.27%	195	38.77%	0.002
Experiences of stigma and violence	Stigma from family and friends (lifetime)	1866	31.33%	1778	30.59%	88	41.60%	<0.01
	General social stigma (lifetime)	2019	33.93%	1931	33.22%	88	61.54%	<0.01
	Anticipated healthcare stigma (lifetime)	818	13.75%	766	13.18%	52	36.36%	<0.01
	Physical assault (lifetime)	661	11.17%	624	10.74%	37	25.87%	<0.01
	Coercive sex (lifetime)	506	8.52%	474	8.18%	32	22.38%	<0.01
	Fear of being in public (lifetime)	655	11.10%	611	10.59%	44	31.88%	<0.01

Of the 18 621 responses, n=16 responses were missing in the sexual orientation question.

* Among those who had anal sex in the past 30 days.

† Combining sex with both non-injectable and injectable drugs.

‡ Of those who disclosed their positive HIV status.

§ Fisher’s exact test of significance.

¶ Of those who ever tested positive for HIV and registered at AIDS centre.

CMSM, cisgender men who have sex with men; NGO, non-governmental-organisation; TGWSM, transgender women who have sex with men.

Table 1 presents differences between sexual and drug-related behaviours between CMSM and TGWSM. Compared with CMSM, a greater proportion of TGWSM provided commercial sex in their lifetime (15.17% vs 18.49%, p=0.004) and were involved in group sex (19.9% vs 27.6%, p=0.01). Likewise, TGWSM had a higher mean number of long-term sexual partners in the past 30 days than CMSM (0.8 vs 0.7, p=0.04), mean number of partners they paid for sex services (0.08 vs 0.05, p=0.05) and had chemsex in the last 30 days (7.77% vs 4.7%, p=0.001). Lastly, more TGWSM received free condoms in the previous 12 months (58.65% vs 48.76%, p<0.001) and were clients of NGOs (38.77% vs 32.27%, p=0.002) than CMSM, as presented in table 1.

### Demographic characteristics and HIV risk behaviours and care outcomes associated with being transgender

Table 2 illustrates associations between transgender status and risky sexual behaviours, HIV positivity status and engagement with care. After adjusting for demographic characteristics (adjusted OR (aORs) for these demographic variables are presented in online supplemental table 2), identifying as transgender was associated with greater odds of having a prior history of imprisonment (aOR=1.51, 95% CI 1.00 to 2.31), being a client of NGOs (aOR=1.39, 95% CI 1.15 to 1.67), having sexual intercourse with male partner in the last 30 days (aOR=1.48, 95% CI 1.23 to 1.35), providing commercial sex in past 30 days (aOR=1.28, 95% CI 1.01 to 1.61), engaging in group sex (aOR=1.31, 95% CI 1.06 to 1.61) and chemsex in last 30 days (aOR=1.58, 95% CI 1.12 to 2.23). Identifying as transgender was also associated with having a higher number of long-term sex partners in the past 30 days (adjusted incidence rate ratio=1.14, 95% CI 1.03 to 1.27).

**Table 2:** Unadjusted and adjusted associations between transgender status (vs CMSM) and HIV status, linkage with HIV care and treatment, and risk behaviours

	Unadjusted OR* (95% CI)			Adjusted OR* (95% CI)		
	OR	95% CI	P value	OR	95% CI	P value
Tested for HIV (last 12 months)	1.23	0.95 to 1.59	0.106	1.16	0.90 to 1.51	0.235
HIV positive†	0.97	0.65 to 1.45	0.889	1.00	0.65 to 1.50	0.964
Client of an NGO	1.34	1.11 to 1.61	0.002	1.39	1.15 to 1.67	<0.001
Registered at an AIDs Centre	0.32	0.03 to 3.27	0.340	0.34	0.03 to 3.67	0.376
On antiretroviral therapy	1.01	0.07 to 14.2	0.992	0.76	0.05 to 11.2	0.842
Received free condoms	1.48	1.23 to 1.77	<0.001	0.94	0.66 to 1.78	0.766
Sexual intercourse with male partner (6 months)	0.97	0.67 to 1.39	0.870	1.48	1.23 to 1.35	<0.001
Used condom for last anal sex (recent)	0.92	0.75 to 1.13	0.445	0.91	0.74 to 1.12	0.382
Always used condom for last sex (30 days)	1.16	0.93 to 1.44	0.173	1.14	0.91 to 1.42	0.239
Commercial sex procured	1.75	1.16 to 2.66	0.008	1.24	0.82 to 1.88	0.296
Commercial sex provided	2.49	2.04 to 3.03	<0.001	1.28	1.01 to 1.61	0.034
Group sex (with men and women)	1.28	1.04 to 1.58	0.016	1.31	1.06 to 1.61	0.011
Chemsex in last 30 days (non-injectable drugs)	1.65	1.18 to 2.32	0.003	1.58	1.12 to 2.23	0.008
Number of long-term anal sex partners (30 days)‡	1.12	1.01 to 1.25	0.025	1.14	1.03 to 1.27	0.010
Number of casual anal sex partners (30 days)‡	1.12	0.97 to 1.29	0.108	1.07	0.93 to 1.24	0.290
History of imprisonment	1.48	0.99 to 2.22	0.054	1.51	1.00 to 2.31	0.049

Adjusted OR and CI values for age and higher education are presented in online supplemental materials.

* Adjusted and unadjusted OR from mixed-effects logistic regression with city and survey year as the crossed random effects.

† Tested positive for HIV.

‡ Incidence-rate ratios from mixed-effects negative binomial regression with city and survey year as the crossed random effects.

CMSM, cisgender men who have sex with men; NGO, non-governmental-organisation.

Identifying as transgender was not significantly associated with testing positive for HIV in past 12 months, Being HIV positive, being registered at an AIDS centre, being on ART, using a condom for last anal sex and having more casual sex partners.

### Comparing lifetime experience of stigma and violence between TGWSM and CMSM

Table 1 illustrates the differences in lifetime experiences of stigma and violence faced by TGWSM and CMSM in the 2018 survey. Compared with CMSM, a much greater proportion of TGWSM reported facing different forms of stigma, including stigma from family and friends (41.60% vs 30.59%, p<0.001), general social stigma (61.54% vs 33.22%, p<0.001), anticipated healthcare stigma (36.36% vs 13.18%, p<0.001), physical assault (25.87% vs 0.74%, p<0.001) and coercive sex (22.38% vs 8.18%, p<0.001). Also, more TGWSM (32.88%) reported they were fearful of being in the public than CMSM (10.59%). Demographic characteristics, sexual behaviours and engagement with care details for all MSM from the 2018 Survey are given in online supplemental table 3.

### Characteristics associated with lifetime experience of stigma and violence

Table 3 presents the adjusted and unadjusted ORs for experiences of stigma by population demographic characteristics and transgender status. After adjusting for age, higher education and imprisonment history, TGWSM were more likely to report stigma from family and friends (aOR=3.58, 95% CI 2.54 to 5.04), general social stigma (aOR=3.13, 95% CI 2.22 to 4.41), anticipated healthcare stigma (aOR=3.63, 95% CI 2.55 to 5.16), physical assault (aOR=2.73, 95% CI 1.85 to 4.03), coercive sex (aOR=3.01, 95% CI 1.99 to 4.55) and being fearful of being in public due to their sexual orientation (aOR=3.74, 95% CI 2.58 to 5.40).

**Table 3:** Unadjusted and adjusted ORs for experiences of stigma and violence for transgender identity, HIV positive status and demographic characteristics

	Adjusted OR (95% CI and p value)														
	Transgender			HIV positive			Age (per year)			History of Imprisonment (Ref: no history)			Higher education* (Ref: below degree-level)		
Stigma from family and friends†	3.58	2.54 to 5.04	<0.01	0.99	0.77 to 1.28	0.963	1.00	0.98 to 1.00	0.122	1.96	1.45 to 2.64	<0.01	1.03	0.91 to 1.17	0.605
General social stigma†	3.13	2.22 to 4.41	<0.01	1.38	1.08 to 1.74	0.02	1.00	0.98 to 1.00	0.26	1.95	1.44 to 2.62	0.00	0.93	0.82 to 1.05	0.01
Anticipated healthcare stigma†	3.63	2.55 to 5.16	<0.01	1.42	1.05 to 1.92	0.01	1.00	0.99 to 1.01	0.11	2.02	1.42 to 2.85	<0.01	0.79	0.66 to 0.93	<0.01
Physical assault†	2.73	1.85 to 4.03	<0.01	1.19	0.83 to 1.69	0.350	0.99	0.97 to 0.99	0.002	3.92	2.80 to 5.48	<0.01	0.93	0.77 to 1.12	0.456
Coercive sex†	3.01	1.99 to 4.55	<0.01	1.45	1.00 to 2.10	0.049	0.99	0.98 to 1.00	0.308	4.38	3.09 to 6.20	<0.01	0.77	0.61 to 0.95	0.018
Fear of being in public†	3.74	2.58 to 5.40	<0.01	1.09	0.75 to 1.56)	0.652	0.99	0.98 to 1.00	0.092	1.62	1.08 to 2.44	0.019	0.78	0.64 to 0.95	0.014

N=5971 MSM and transgender women from 2018 survey year.

* Higher education included degree-level qualifications from completed undergraduate to doctorate level.

† These experiences were faced due to the individual having sex with men.

MSM, men who have sex with men.

Overall (ie, among both TGWSM and CMSM), HIV prevalence was higher among participants who had previously been exposed to general social stigma (aOR=1.38, 95% CI 1.08 to 1.74), anticipated healthcare stigma (aOR=1.42, 95% CI 1.05 to 1.92), and coercive sex (aOR=1.45, 95% CI 1.00 to 2.10). History of imprisonment positively correlated with stigma from family and friends (aOR=1.96, 95% CI 1.45 to 2.64), general social stigma (aOR=1.96, 95% CI 1.45 to 2.64), anticipated healthcare stigma (aOR=2.02, 95% CI 1.42 to 2.85), physical assault (aOR=3.92, 95% CI 2.89 to 5.48) and coercive sex (aOR=4.38, 95% CI 3.09 to 6.20). The victims of physical assault (lifetime exposure) were also more likely to be of younger age (aOR=0.99, 95% CI 0.98 to 0.99), presented in table 3. The differences in predicted probabilities between transgender participants and CMSM for experiencing each stigma and violence outcome are presented in online supplemental table 4. The probabilities that transgender participants had experienced stigma from family and friends or general social stigma were 31% and 22% more (in absolute terms) than for CMSM. Similarly, the probabilities of experiencing physical or sexual violence were 13%–14% higher for transgender participants than those for CMSM.

## Discussion

This study examines the disparities in sexual risk behaviours, HIV prevalence, HIV treatment uptake and prevention engagement between TGWSM and CMSM in Ukraine. The findings reveal higher levels of engagement in sexual and drug-related behaviours such as engaging in commercial sex, having multiple partners and participating in chemsex, among TGWSM compared with CMSM. TGWSM also demonstrate greater involvement with NGOs offering HIV prevention, testing and treatment linkage services, but there were no significant differences in condom use, HIV testing or treatment outcomes.

Our findings demonstrated that CMSM and TGWSM both experienced high levels of stigma and violence—experiences which are thought to affect HIV risk behaviours and access to HIV prevention and care^30^,^34^ and were associated with being HIV positive in our study. However, TGWSM were approximately three times more likely to experience stigma and violence than CMSM. Research has highlighted that transphobia is embedded in cisnormativity and heteropatriarchal societal norms^7 9^ and operates on multiple levels, including structural and interpersonal factors, which play a critical role in shaping the health-related vulnerabilities, access to care and prevention among transgender persons.^9^ In our study, more than a third of transgender participants reported being fearful of seeking healthcare, while 20% avoided seeking healthcare due to this fear. Such high levels of anticipated healthcare stigma may not only significantly impact on engagement with HIV care among transgender individuals but also their wider health and highlights the need for culturally sensitive care for transgender populations.

We also found that transgender participants were more likely to have a history of imprisonment than CMSM, which may indicate disproportionate policies and structural barriers to transgender people’s rights in communities. The intersection of transphobia, marginalisation and structural limitations to transgender rights, in parallel with economic vulnerabilities that arise from these societal barriers, may explain these increased levels of incarceration. The same structural barriers can negatively influence opportunities in education, housing, employment, right to marry and gender affirmation.^35 36^ These economic challenges that transgender persons face in society may explain the increased levels of commercial sex among TGWSM. There is a need for programmes and policies that ensure social protection and equal opportunities for transgender and other sexual minority populations in education, employment and access to healthcare.

While we found greater levels of risk and high-risk behaviours among transgender participants than cisgender participants, we did not demonstrate a statistically significant difference in HIV prevalence between the two population groups. This could be attributed to the low number (about 5%) of HIV positive individuals in each group limiting the statistical power. Indeed, previous studies have consistently demonstrated a strong correlation between risk and prevention behaviours and HIV positivity in MSM populations across various geographies.^37^,^40^

This study used data from three national IBBS of MSM in Ukraine, comprising data from over 18 000 respondents. The data included information about HIV testing and treatment, risk behaviours, stigma and other negative experiences, as well as demographic information of the respondents, enabling us to explore behavioural practices, service utilisation patterns and experiences of stigma faced by TGWSM and CMSM. The geographic representation of all regions of Ukraine and the large sample size increases the generalisability of the study findings. However, approximately 90% of the survey participants were below the age of 40, substantially higher than the general population demographics (45% adult males <40 years). The underrepresentation of older MSM and TGW in these surveys is a limitation of this data, as commonly found in MSM surveys,^41^,^43^ and may limit the generalisability of the findings to older MSM and transgender populations. Moreover, as the participants in this study were recruited using the RDS methodology using chain referral sampling, the study findings cannot be generalised to the overall MSM populations across Ukraine and representative of the population groups reached through IBBS recruitment mechanisms.

Another limitation of this study is the transgender participants in the IBBS were involved through NGOs. Consequently, the findings pertaining to the engagement of transgender women with NGOs may not accurately reflect the broader patterns of NGO engagement in Ukraine. Moreover, there was a lack of questions on stigma and related negative experiences in earlier surveys, and so only data from 2018 was used to measure these outcomes. Responses to many questions about risk behaviours and experiences of stigma and violence may be subject to recall bias, as with other self-reported behavioural surveys. Particularly, experiences of stigma, violence and victimisation may be discomforting to recall, due to which participants may have avoided expressing these experiences. Additionally, social desirability or acquiescence bias due to stigma associated with homosexual partnerships, condomless intercourse and chemsex is likely, as the participants may feel hesitant to report behaviours that they consider unacceptable to the society. On the other hand, behaviours such as HIV testing trends and PrEP use are likely to be over-reported. However, it is unlikely that these biases would influence the responses of transgender and CMSM differently. Stigma towards sexual minorities is also widespread, which could have influenced MSM’s ability to participate in the survey or express their true gender identity or sexual orientation in the survey.

Another challenge in this study was the use of the term ‘men who have sex with men’ for all study participants, which in the case of transgender persons is unsuitable. We have used the term transgender women who have sex with men throughout. However, the sensitivity and specificity of the IBBS survey instrument to distinguish between nuanced transgender identities or gender-diverse categories has not been established in detail, likely resulting in some misclassification and inclusion of other gender-diverse individuals within the category of TGWSM. Similarly, items within the stigma instrument, which have been validated among MSM in the US and Sub-Saharan Africa but not Eastern Europe, were specific to homosexuality-based stigma (androphilic sexual behaviours and male-assigned status). As such, the significant levels of stigma and violence found among transgender participants within our study likely underestimate the broader experiences of stigma among transgender participants.

Finally, the findings of this study present an overview of the vulnerabilities of MSM and TGW in Ukraine where stigma, discrimination and violence against sexual and gender-minority individuals is more prevalent than some other more progressive regions in Europe. As such, our findings may not be generalisable to other regions in Central or Western Europe, or settings outside Europe, where sociocultural contexts and levels of stigma differ considerably. While further research is needed to establish causal links between stigma and violence and HIV risk among MSM and transgender populations, it is important that this occurs across countries with differing sociocultural and legal and policy environments to better inform policy and programming for HIV prevention among MSM and TGW populations.

## Conclusions

The present study substantiated that transgender women and MSM have differing levels of HIV risk, with transgender women having higher levels of sexual risk, while their needs are largely unmet due to the continued approach of treating MSM and TGW alike in public health research, policy and practice. The role of NGOs in the prevention and treatment of HIV/AIDS in Ukraine was pivotal before the start of the war;^44^ however, stigma persists and needs to be addressed to improve HIV treatment and prevention efforts targeted for CMSM and TGWSM. Although TGWSM were more likely to engage with NGOs than CMSM, their higher prevalence of high-risk behaviours suggests that these HIV prevention services may need to be adapted for this group to further help them manage high-risk behaviours. This could include making programmes more trans-inclusive that are responsive to the sexual health and psychological needs of transgender and other gender minority populations. Inclusion of stigma and harm reduction interventions in national HIV prevention programmes could be crucial to enhance access to HIV services for sexual minorities. Services specifically tailored for transgender people, such as improving provider competency, tackling healthcare stigma and enhancing community-based outreach services^45^ are needed to help curtail the negative effects of stigma on HIV prevention and care. Directing additional attention towards health literacy and comprehensive sexual health education programmes could be important in reducing risky sexual practices and improving HIV testing in these populations. Lastly, we also recommend adoption of ‘gender identity’ as a construct to distinguish persons of different gender minorities in HIV monitoring and programme and policy planning instead of referring to ‘biological sex’ of transgender communities.

## Supplementary material

10.1136/bmjopen-2025-104918 online supplemental file 1

## Data Availability

Data may be obtained from a third party and is not publicly available.
