# Supplementary material for: Differences in sexual risk behaviours, HIV care utilisation and experiences of stigma between transgender women and cisgender men who have sex with men: findings from integrated biobehavioural surveys in Ukraine 2013–2018
Source: BMJ Open. 2026 Feb 10;16(2):e104918. doi: 10.1136/bmjopen-2025-104918 (PMC12911835; doi:10.1136/bmjopen-2025-104918)
Supplement: online supplemental file 1 [file bmjopen-16-2-s001.docx]

## Supplementary Materials

**Table 1- Measurement of Transgender Status in IBBS Surveys**

| IBBS Year | Inclusion criteria and definition of MSM | Item on Transgender Status | Response Options | Coding Rule |
| --- | --- | --- | --- | --- |
| 2013, 2016, and 2018 | Biological men who had during the last 6 months at least one oral or anal sexual contact with another biological male partner. This group includes, inter alia, transgender people who are biologically male and practice sex with men. | “Do you consider yourself as transgender?” | Yes / No | All respondents who answered “Yes” were coded as transgender. |

**Table 2 - Details of unadjusted and adjusted odds ratio for Transgender, HIV positive status, linkage with service for demographic characteristics and risk behaviours.**

|  | | | | | |  |
| --- | --- | --- | --- | --- | --- | --- |
| **Adjusted OR (95% Confidence Interval)** | | | | |  |  |
|  | **Age** | | ***Higher Education*** | |  |  |
| Tested for HIV (last 12 months) | 0.97 | (0.96 – 0.98) | 1.05 | (0.96 - 1.15) |  |  |
| HIV positive † | 1.00 | (0.99 - 1.01) | 1.17 | (1.02 - 1.35) |  |  |
| Client of an NGO | 1.00 | (1.00 - 1.01) | 1.22 | (1.13 - 1.30) |  |  |
| Registered at an AIDs Centre | 1.03 | (0.99 - 1.08) | 1.49 | (0.64 - 3.48) |  |  |
| On antiretroviral therapy | 1.02 | (0.98 - 1.06) | 2.25 | (1.08 - 4.67) |  |  |
| Sexual intercourse with male partner (6 months) | 0.99 | (0.99 – 1.00) | 0.93 | (0.81 - 1.05) |  |  |
| Received free condoms | 1.00 | (0.99 - 1.00) | 1.12 | (1.05 - 1.19) |  |  |
| Used condom for last anal sex (30 days) | 0.99 | (0.99 - 1.00) | 1.01 | (0.94 - 1.09) |  |  |
| Always used condom for last sex (30 days) | 0.99 | (0.99 - 1.00) | 0.89 | (0.82 - 0.96) |  |  |
| Commercial sex procured | 1.05 | (1.04 - 1.06) | 1.67 | (1.39 - 2.00) |  |  |
| Commercial sex provided | 0.98 | (0.97 - 0.98) | 0.60 | (0.54 - 0.66) |  |  |
| Group sex (men & women) | 0.99 | (0.99 - 1.00) | 1.08 | (1.00 - 1.17) |  |  |
| Chemsex in last 30 days (non-injectable drugs) | 0.96 | (0.95 - 0.97) | 0.87 | (0.74 - 1.03) | |  |
| Mean number of long-term anal sex partners (30 days) †* | 1.00 | (1.001 – 1.005) | 1.04 | (1.00 - 1.08) | |  |
| Mean number of casual anal sex partners (30 days) †* | 0.99 | (0.98 – 0.99) | 0.92 | (0.87 - 0.97) | |  |
| History of imprisonment | 1.06 | (1.06 – 1.07) | 0.41 | (0.34 – 0.50) |  |  |
| † Tested for HIV.  †*Incidence-rate ratios. | | | | |  |  |

**Table 3 - Demographic characteristics, sexual behaviours, and engagement with care details for all participants from 2018 Survey (n=5,971).**

|  | | |
| --- | --- | --- |
| Variable | | percentages |
| Demographics | Mean Age | 28.9 years |
|  | History of Imprisonment | 3.18% |
|  | Higher Education | 31.76% |
|  | Transgender | 2.40% |
|  | HIV test result | 5.23% |
| Sexual Practices & behaviours | Provided commercial anal sex for money (lifetime) | 16.95% |
|  | Provided commercial anal sex for money (30 days) | 26.82% |
|  | Bought commercial anal sex (30 days) | 2.50% |
|  | Group sex (6 months) | 17.16% |
|  | Group sex with men (6 months) | 13.90% |
|  | Group sex with women (6 months) | 0.30% |
|  | Group sex with both men and women (6 months) | 17.16% |
|  | Sex with a woman/FSW (6 months) | 4.46% |
|  | Mean number of total anal sex partners (30 days) | 2.34 |
|  | Mean number of long-term anal sex partners (30 days) | 1.4 |
|  | Mean number of casual anal sex partners (30 days) | 3.2 |
|  | Mean number of partners they paid for anal sex (30 days) | 1.6 |
|  | Mean number of partners who paid them for anal sex (30 days) | 3.17 |
| Chemsex | Chemsex in last 30 days (non-injectable drugs) | 6.95% |
|  | Chemsex in last 30 days (injectable drugs) | 23.81% |
| Use of Condoms | Received free condoms (12 months) | 44.15% |
|  | Bought condoms (30 days) | 35.28% |
|  | Used condom for anal sex (30 days) | 77.39% |
|  | Used condom for anal sex with casual partner (30 days) | 87.63% |
|  | Used condom for anal sex with long-term partners (30 days) | 70.43% |
|  | Used condom for anal sex with commercial sex partners (30 days) | 85.81% |
|  | Used condom for anal sex when provided commercial sex (30 days) | 74.06% |
|  | Used condom for sex with women (30 days) | 67.25% |
| Health-seeking Behaviours | Ever tested for HIV | 65.41% |
|  | Tested for HIV last 12 months | 84.99% |
|  | Registered at an AIDS centre | 91.89% |
|  | Client of an NGO | 28.20% |
|  | On antiretroviral therapy | 92.65% |

**Table 4: Predicted probabilities of the outcomes for cisgender and transgender status and the differences in probability for behaviours and stigma and violence.**

| **Outcome** | **Cisgender probability**  **(95% CI)** | **Transgender probability**  **(95% CI)** | **Difference probability**  **(95% CI)** |
| --- | --- | --- | --- |
| **Behaviours** | | | |
| NGO client | 0.336 (0.276–0.403) | 0.413 (0.334–0.497) | 0.077 (-0.069–0.222) |
| Sexual intercourse with male partner (6 months) | 0.934 (0.882–0.964) | 0.930 (0.866–0.965) | -0.003 (-0.097–0.083) |
| Commercial Sex Provided | 0.148 (0.129–0.171) | 0.178 (0.140–0.223) | 0.030 (-0.031–0.095) |
| Chemsex in last 30 days (non-injectable drugs) | 0.029 (0.010–0.080) | 0.045 (0.015–0.127) | 0.016 (-0.065–0.117) |
| History of Imprisonment | 0.025 (0.021–0.029) | 0.037 (0.024–0.057) | 0.012 (-0.005–0.036) |
| **Stigma and violence** | | | |
| Stigma from Family & Friends | 0.306 (0.294–0.319) | 0.615 (0.532–0.692) | 0.309 (0.213–0.398) |
| General Social Stigma | 0.126 (0.117–0.135) | 0.345 (0.272–0.427) | 0.220 (0.137–0.311) |
| Anticipated Healthcare Stigma | 0.328 (0.315–0.341) | 0.607 (0.524–0.684) | 0.279 (0.182–0.369) |
| Physical assault | 0.105 (0.096–0.113) | 0.247 (0.183–0.324) | 0.142 (0.070–0.228) |
| Coercive Sex | 0.076 (0.069–0.084) | 0.204 (0.147–0.277) | 0.128 (0.062–0.208) |
| Fear of being in Public | 0.101 (0.093–0.110) | 0.298 (0.228–0.379) | 0.197 (0.118–0.286) |

**Table 5 – Differences in measures of stigma between TGWSM and CMSM.**

| **Stigma Categories** | **Stigma-related questions in the IBBS Survey** | **Total** | **TGWSM** | **CMSM** |  |
| --- | --- | --- | --- | --- | --- |
|  |  | Percentage | Percentage | Percentage | P value |
| Stigma from family and friends | Not invited to families’ parties because you have sex with men | 9.26% | 25.35% | 8.85% | <0.01 |
| Stigma from family and friends | friends turned away from you because you have sex with men | 21.10% | 43.53% | 20.53% | <0.01 |
| General social stigma | You are blackmailed because you have sex with men | 6.75% | 14.69% | 6.53% | <0.01 |
| General social stigma | You were physically injured (pushed, beaten by hands, or kicked, strangled, etc.) because you have sex with men | 11.17% | 25.87% | 10.74% | <0.01 |
| General social stigma | You think that the incidence of such physical harm was due to the fact that they are MSM because you have sex with men | 82.03% | 88.89% | 81.64% | 0.271 |
| General social stigma | Ever made sexual contacts contrary to your desire because you have sex with men | 8.52% | 22.38% | 8.18% | <0.01 |
| General social stigma | You think that the cases of such sexual abuse were related to the fact that they are MSM | 71.10% | 90.32% | 69.71% | 0.014 |
| Anticipated healthcare stigma | afraid to seek medical help because you have sex with men | 12.54% | 32.87 | 12.03 | <0.01 |
| Anticipated healthcare stigma | did not go to health care facilities because you have sex with men | 7.78% | 19.58% | 7.49% | <0.01 |
| General social stigma | You were afraid to be in public because you have sex with men | 11.10% | 31.88% | 10.59% | <0.01 |
| General social stigma | You were offended because you have sex with men | 27.41% | 44.37% | 26.94% | <0.01 |
| Stigma from family and friends | Family members made discriminatory remarks or gossip about you | 18.37% | 40.85% | 17.78% | <0.01 |
| General social stigma | heard how medical staff have discussed you (gossiping) because you have sex with men | 4.95% | 19.01% | 4.58% | <0.01 |
| General social stigma | Police refused to defend you because you have sex with men | 2.82% | 11.35% | 2.60% | <0.01 |
| General social stigma | felt that you were poorly treated in health care facilities because you have sex with men | 12.54% | 16.20% | 3.63% | <0.01 |

**Table 6: Stigma-related items in the IBBS Survey with the consolidate categories.**

|  | 1. Yes | 2. No | Don’t know |
| --- | --- | --- | --- |
| D1. You haven’t been invited to families’ parties because you have sex with men | 1 | 2 | 3 |
| D2. Family members made discriminatory remarks or gossip about you because you have sex with men. | 1 | 2 | 3 |
| D3. Your friends turned away from you because you have sex with men | 1 | 2 | 3 |
| D4. You were afraid to seek medical help because someone could find out that your sexuality is aimed at men | 1 | 2 | 3 |
| D5. You did not go to health care facilities because someone can find out that you have sex with men. | 1 | 2 | 3 |
| D6. You felt that you were poorly treated in health care facilities because you knew that you had sex with men | 1 | 2 | 3 |
| D7. You have heard how medical staff have discussed you (gossiping) because your sexuality is aimed at men | 1 | 2 | 3 |
| D8. Police refused to defend you because you have sex with men | 1 | 2 | 3 |
| D9. You were afraid to be in public because of the fact that you have sex with men | 1 | 2 | 3 |
| D10. You were offended because you have sex with men | 1 | 2 | 3 |
| D11. You are blackmailed because you have sex with men | 1 | 2 | 3 |
| D12. You were physically injured (pushed, beaten by hands, or kicked, strangled, etc.) because you have sex with men | 1 | 2 🡪 D14 | 3 🡪 D14 |
| D13. Do you think that the incidence of such physical harm was due to the fact that you have sex with men? | 1 | 2 | 3 |
| D14. Have you ever made sexual contacts contrary to your desire (under coercion, we mean physical coercion to have sex with a person or subjects when you did not want to)? | 1 | 2🡪 E1 | 3🡪 E1 |
| D15. Do you think that the cases of such sexual abuse were related to the fact that you have sex with men? | 1 | 2 | 3 |
| Items D1, D2 and D3 were collated to form the category “Stigma from family and friends”.  Items D4 and D5 were categorised as “Anticipated healthcare stigma”.  Items D6 – D15 were categorised as “General social stigma”. | | | |
