# Supplementary material for: Differences in sexual risk behaviours, HIV care utilisation and experiences of stigma between transgender women and cisgender men who have sex with men: findings from integrated biobehavioural surveys in Ukraine 2013–2018
Source: BMJ Open. 2026 Feb 10;16(2):e104918. doi: 10.1136/bmjopen-2025-104918 (PMC12911835; doi:10.1136/bmjopen-2025-104918)
Supplement: Supplementary file 2 [file bmjopen-2025-104918.reviewer_comments.pdf]

PEER REVIEW HISTORY

BMJ Open publishes all reviews undertaken for accepted manuscripts. Reviewers are asked to complete a checklist review form and are provided with free text boxes to elaborate on their assessment. These free text comments are reproduced below.

ARTICLE DETAILS

Title (Provisional)

Differences in sexual risk behaviours, HIV care utilisation, and experiences of stigma between transgender women and cisgender men who have sex with men: findings from Integrated Biobehavioural Surveys in Ukraine 2013 – 2018.

Authors

Aijaz Khan, Saher; Vickerman, Peter; Saliuk, Tetiana; Nicholls, Jane; Gillespie, David; Hood, Kerenza; Stone, Jack

---

VERSION 1 - REVIEW

**Reviewer**	**1**
**Name**	**Skeen, Simone**
**Affiliation**	**Tulane University**
**Date**	**30-Jul-2025**
**COI**	**None**

General comments to author

The submitted manuscript pools Integrated HIV biobehavioral survey (IBBS) data collected in Ukraine across 2013, 2015, and 2018, revealing a range of structural/systemic, psychosocial, and HIV prevention and treatment cascade disparities between cisgender and transgender survey respondents. These disparities are stark, and point to urgent, unmet, needs among the presumed-AMAB trans respondents of whom the analytic sample is composed. From a health equity perspective, the merit of these analyses is unquestionable. But, in the paper's present state, the fundamental limitations of the IBBS instrumentation in capturing respondent-level SO/GI information, at an appropriate level of sensitivity and specificity to support the framing of these results, remains under-acknowledged. Chief among these shortcomings is the lack of a reported gender identity variable, which renders the interpretation of AMAB (or "biological male," a term that should be eschewed) + "transgender" respondents as "transgender women" across the board dubious. A similar issue emerges concerning the interpretation of the stigma items. This critique should not be read as undermining the motivation to highlight these findings; indeed, broadening the narrative lens to recognize the likely heterogeneity within the transgender sample is more

closely aligned with e.g. UNAIDS key population definitions, and should not necessitate a total analytic overhaul at R1s.

The considerable across-case heterogeneity that is likely encoded within the "TGWSM" sample calls for a major revision, in framing and narrative interpretation, if not in analytic approach (though see my recommendations regarding marginal effects). Grounding the research within appropriate (and summative) conceptual frameworks of HIV disparity among trans populations such as Sevelius's or Reisner's will stabilize the presently patchy mis/use of terms such as "MSM" and "biological male," while attaching the present work to a lineage of global HIV equity research, more broad than the presently inaccurate "transgender women" focus.

Specific comments

Abstract

Note: the manuscript PDF contains inconsistent pagination. I refer to the running-header "Page n of N" pagination throughout.

Pages 2–3 of 33: It is a bit disjoint to use "TG" to abbreviate "trangender" and "C" to abbreviate "cisgender." I recommend using the more intuitive TWSM and CMSM, respectively.

Page 3 of 33: "(2013/15/18)" is a confusing notation; "(2013, 2015, and 2018)" would be clearer.

Key Messages

Page 4 of 33: "What is already known on this topic: Men who have sex with men and transgender women are at high risk of HIV." Not every trans woman is at high risk of acquiring HIV. As we are in a global epi versus social epi/biobehavioral HIV science domain, this statement should be qualified accordingly: "Men who have sex with men and transgender people are recognized by [PEPFAR/UNAIDS/etc.] as key populations at elevated risk of HIV acquisition."

Introduction

Page 5-6 of 33: "HIV transmission in this group, like other KPs, is marked by involvement in high-risk sexual behaviours and practices including condomless sex, transactional sex, and injectable and non-injectable drug use [9 10]. These behaviours and practices intersect with other individual and societal level factors such as mental health and other health-related comorbidities, stigma, discrimination, and persecution [7 11]. Similarly, social challenges arising from structural barriers, such as limited chances for higher education or employment opportunities and lack of recognition of transgender persons' gender by society, can also play a role in increasing the transmission of HIV within these populations, by limiting access to resources that promote health and protection [7 11 12]." This passage attempts to describe causal factors of disease (mal)distribution among trans people, acknowledging

multilevel and interlocking drivers of sexual decision-making patterns to which we ascribe “risk” given the ongoing HIV epidemic. Presently, it lacks focus, portraying behaviors such as CAS and phenomena such as structural transphobia as occurring in parallel, rather than in an entangled, syndemic, manner. As this passage is meant to motivate the analysis, succinctly, I recommend grounding it in a cohesive theory of HIV disparity among trans people. The most comprehensive such framework is Reisner’s Situated Vulnerabilities and Resiliencies Framework (Reisner, 2025; https://doi.org/10.1016/S2352-3018(24)00299-6), a simple summary of which would accomplish more than this paragraph in its present state.

Page 5 of 33: The “KP” abbreviation is not used consistently, and the term itself occurs sporadically; it is not necessary to abbreviate. As well, precisely which standard-setting body (UNAIDS, PEPFAR, etc.) is defining “key populations” in the manner it is used in this paper should be stated explicitly.

Page 6 of 33:

“Infection” is considered stigmatizing in contemporary HIV science. “Acquisition” should be used here (line 26).

“IBBS” is never expanded throughout the manuscript. Given that this paper will likely be of interest to a transdisciplinary readership of health disparities and SGM health researchers, the Integrated Biological and Behavioral Surveillance approach should be very briefly defined.

References to the original IBBS reports presently lack URLs, and they are difficult to retrieve, lacking distinct identifiers. Please add the appropriate URLs.

Page 7 of 33:

“[...] biological men, who self-reported sexual contact (oral or anal) with another man in the previous six months” is inaccurate here; aspects of medically affirmed trans women’s “biology” more closely align with that of cis women’s, and the total exclusion of trans men cannot be assumed given the IBBS methodology (as reported here). As birth-assigned sex is unlikely to have been ascertained in the midst of an RDS protocol, the more precise term would be “presumed assigned male at birth.” As the sample under consideration here is presumed trans women, “another man” should be replaced with “a man.”

“Transgender status was characterised using responses to the question ‘Do you consider yourself as transgender’.” The presumption of a birth-assigned male sex and a “yes” endorsement to this item is sensitive enough, but not specific enough, to reliably encode every respondent who identifies as a woman, trans woman, or person on the trans-feminine spectrum. Indeed, it could as easily capture a nonbinary or questioning individual, or a trans man. While it is unsurprising that older population surveys in high-stigma contexts would fall short of contemporary SO/GI harmonization best practices, this limitation of the IBBS instrument, and the ambiguities in interpretation it introduces, must be acknowledged. Given that PEPFAR, UNAIDS, and the WHO recognize transgender and gender-diverse

people, more broadly, as a population disproportionately burdened by HIV, the manuscript retains its value by more precisely characterizing the comparisons being reported here as transgender versus cisgender individuals who have sex with men, acknowledging that that supercategory definitionally includes TWSM in Ukraine, but may overrepresent the (presently unknowable) risk markers specific to TWSM in Ukraine.

I recommend providing the precise language of the “sex”-based inclusion criteria, and transgender status item, for each IBBS pooled here, in a text box or small table perhaps. Narratively, “transgender people” or “individuals” should be favored, with “transgender respondents, including trans women” used when discussing factors most salient (theoretically) to structural and interpersonal transmisogyny.

Page 8 of 33:

“To account for potential confounding, we also adjusted our models for age (in years) and higher education attainment (completed undergraduate or higher degrees).” This particular control set should be justified theoretically. The precise operationalizations of these controls – e.g. whether age was entered as FEs, in strata, etc. – should be stated, either here or as a footnote to the appropriate tables.

“These measures were derived from a 15-item inner homophobia scale (Supplementary Tables 3 and 4).” This is another area where interpretation of these findings is muddied by the relative crudity of the IBBS instrumentation. Descriptively, the disparities reported in Supplementary Tables 3 and 4 are striking. But many (not all) of these stigma items are clearly contextualized “...because you have sex with men.” That stigmatization process is, therefore, predominantly in response to the respondents’ trans status and androphilic sexual behaviors. Qualitatively, there is likely a lot of case-by-case heterogeneity obscured by those estimates, including the stigmas particular to TWSM. This point should be covered in the Discussion.

At the same time, there are also powerful findings evident in the items (e.g. “afraid to seek medical help”; “afraid to be in public”) that lack the “because you have sex with men” qualifier. It is worth foregrounding these results, both because interpretation is a bit clearer, and the disparities equally as compelling.

Results

Page 9 of 33:

As BMJ Open is a generalist title, I recommend defining “chemsex” the first time the term is used (outside of the abstract).

a/ORs are notoriously difficult to interpret, and, for the effects the authors feel are most implicative or compelling, should be accompanied by marginal effects. I recommend using Stata’s post-estimation margins commands to provide the practical significance of these findings [e.g. “identifying as transgender was associated with greater odds of having a prior history of imprisonment (aOR=1.51, 95% CI: 1.00–2.31): a 0.18 uptick in probability of

imprisonment relative to cisgender comparators"]. Whether average marginal effects, marginal effects at representative values, etc., are most appropriate, I leave to the authors. (Note that "0.18" is a toy value for illustrative purposes.)

Discussion

Page 12 of 33:

"Transphobia, which is the societal discrimination of Persons whose gender identity and sexual practices do not correspond to societal standards." Transphobia involves the enforcement, via violence, the denial of opportunity, and the rescindment of bodily autonomy, of cishetero-patriarchal norms. It does not involve "sexual practices," focally, and should not be conflated with the specific stigmatization processes that afflict sexually minoritized communities – a point that is raised in Wesley King's review, cited here. (As well, there is a wealth of contemporary research on transphobia and trans-specific vectors of structural stigma; there is no reason to cite an article from 2006 to support this argument.) See my former point regarding grounding these analyses in a cohesive framework such as Reisner's, which will require raising these points (disambiguated from e.g. homophobia) in the Introduction.

In general, I recommend reserving the overview of structural/systemic drivers of HIV disparity, appropriately grounded, for the Introduction. The Discussion would more constructively focus on (actionable) implications, plus strengths and limitations, rather than "big picture" narratives.

**Reviewer**	**2**
**Name**	**Chan, Alex Siu Wing**
**Affiliation**	**The Hong Kong Polytechnic University**
**Date**	**06-Aug-2025**
**COI**	**None**

The study aims to compare sexual risk behaviors, HIV care utilization, and experiences of stigma between transgender women who have sex with men (TGWSM) and cisgender men who have sex with men (CMSM) in Ukraine. After reviewing the manuscript, I believe the study presents a crucial topic and provides important insights into the disparities between these two populations. However, I have a few considerations and suggestions for improvement.

1. Introduction:

The introduction discusses the growing HIV epidemic in Eastern Europe and Central Asia, with a particular focus on Ukraine. For my sense, while the context is well-established, I wonder if the introduction could benefit from a clearer explanation of how the findings

specifically add to the existing literature. I believe the intersection between HIV risk behaviors and stigma could be explored further, particularly how these factors interact with socio-economic challenges. Expanding on these aspects would enhance the understanding of the gaps the study aims to address and potentially draw more attention to the importance of the research.

2. Methods:

In the methods section, the study does a decent job of outlining the design, sampling strategy, and statistical techniques. However, I feel there needs to be further clarification regarding the use of respondent-driven sampling (RDS) and the absence of RDS weighting in the regression models. I am wondering if the rationale for excluding RDS weights could be better explained. Furthermore, the survey instrument’s validity and reliability are not sufficiently addressed. In my view, including a brief discussion on the potential limitations of the methodology would help to strengthen this section. Additionally, clearer details on how transgender participants were categorized in the analysis could improve transparency.

3. Results:

While the results section provides a comprehensive dataset comparing TGWSM and CMSM, I am considering that there could be more discussion on how these behavioral differences may influence broader public health policies and interventions. The results would benefit from being summarized more clearly, particularly concerning HIV prevalence and care engagement. For my focus, I believe the presentation of tables and figures is essential, but clearer labeling and more direct references within the text would improve readability. Additionally, further elaboration on the clinical implications, particularly tailored interventions for TGWSM, would enhance this section.

How does the significant difference in mean age (27.67 for TGWSM vs. 28.83 for CMSM) impact the study's findings on sexual risk behaviors? I'm wondering what might explain the slightly higher proportion of TGWSM (3.26%) with a history of imprisonment compared to CMSM (2.05%). Why is there no significant difference in HIV prevalence (5.17% for TGWSM vs. 5.43% for CMSM)? I’m curious about the underlying reasons for the higher mean number of long-term sex partners in TGWSM (0.8) compared to CMSM (0.7). How do the findings on chemsex (7.77% in TGWSM vs. 4.7% in CMSM) influence HIV prevention strategies? I also wonder what factors could explain the higher percentage of TGWSM receiving free condoms (58.65%) compared to CMSM (48.76%). Why was there no significant difference in the use of condoms for the last anal sex (74.16% for TGWSM vs. 75.80% for CMSM)? How do the results on commercial sex involvement (18.49% in TGWSM vs. 15.17% in CMSM) contribute to the overall risk assessment for HIV? The higher levels of stigma faced by TGWSM (41.60% reported stigma from family and friends vs. 30.59% in CMSM) make me think about their healthcare access—what implications does this have? Finally, how might the difference in fear of being in public (31.88% in TGWSM vs. 10.59% in CMSM) inform public health policies targeting these groups?

4. Discussion:

The discussion does an adequate job of interpreting the findings, but I feel it could offer a more detailed analysis of the social and structural factors contributing to the behaviors observed among TGWSM. After reviewing the manuscript, I think the discussion of stigma is important, but it could go further in addressing the role of institutionalized stigma, especially with regard to healthcare access. It would be helpful to make a more explicit connection between the statistical findings and their real-world implications for HIV prevention strategies. Additionally, comparing these results to similar studies in other regions could provide greater validation and offer a broader perspective on the findings.

5. Conclusion and Limitations:

The conclusion provides a brief summary of the study's findings but lacks a deeper reflection on the broader societal implications. In my opinion, it would be more impactful if it offered more practical recommendations for improving HIV care services specifically tailored to transgender populations. The limitations are mentioned, but for my sense, the potential for recall bias, social desirability bias, and underrepresentation of older individuals could be more explicitly acknowledged. I believe the study's external validity, particularly how generalizable the results are to other regions, needs more consideration. Expanding on how these findings could influence future research on transgender health would provide a valuable direction for subsequent studies.

VERSION 1 - AUTHOR RESPONSE

Reviewer: 1
Dr. Simone Skeen, Tulane University
Comments to the Author:
General comments to author

The submitted manuscript pools Integrated HIV biobehavioral survey (IBBS) data collected in Ukraine across 2013, 2015, and 2018, revealing a range of structural/systemic, psychosocial, and HIV prevention and treatment cascade disparities between cisgender and transgender survey respondents. These disparities are stark, and point to urgent, unmet, needs among the presumed-AMAB trans respondents of whom the analytic sample is composed. From a health equity perspective, the merit of these analyses is unquestionable. But, in the paper's present state, the fundamental limitations of the IBBS instrumentation in capturing respondent-level SO/GI information, at an appropriate level of sensitivity and specificity to support the framing of these results, remains under-acknowledged. Chief among these shortcomings is the lack of a reported gender identity variable, which renders the interpretation of AMAB (or "biological male," a term that should be eschewed) + "transgender" respondents as "transgender women" across the board dubious. A similar issue emerges concerning the interpretation of the stigma items. This critique should not be read as undermining the motivation to highlight these findings; indeed, broadening the narrative lens to recognize the likely heterogeneity within the transgender sample is more closely aligned with e.g. UNAIDS key population definitions, and should not necessitate a total analytic overhaul at R1s.

The considerable across-case heterogeneity that is likely encoded within the "TGWSM" sample calls for a major revision, in framing and narrative interpretation, if not in analytic approach (though

see my recommendations regarding marginal effects). Grounding the research within appropriate (and summative) conceptual frameworks of HIV disparity among trans populations such as Sevelius's or Reisner's will stabilize the presently patchy mis/use of terms such as "MSM" and "biological male," while attaching the present work to a lineage of global HIV equity research, more broad than the presently inaccurate "transgender women" focus.

Specific comments

Abstract

Comment: Note: the manuscript PDF contains inconsistent pagination. I refer to the running-header "Page n of N" pagination throughout.
Response: We have updated the page numbers.

Comment: Pages 2–3 of 33: It is a bit disjoint to use "TG" to abbreviate "transgender" and "C" to abbreviate "cisgender." I recommend using the more intuitive TWSM and CMSM, respectively.

Response: We chose to use the abbreviation TGWSM (transgender women who have sex with men) because it is more consistent with terminology commonly used in the public health and HIV literature (included those by UNAIDS authors – Korenkomp et al.)

Korenromp EL, Sabin K, Stover J, Brown T, Johnson LF, Martin-Hughes R, Ten Brink D, Teng Y, Stevens O, Silhol R, Arias-Garcia S. New HIV infections among key populations and their partners in 2010 and 2022, by world region: a multisources estimation. JAIDS Journal of Acquired Immune Deficiency Syndromes. 2024 Jan 1;95(1S):e34-45.

Scott HM, Spinelli M, Vittinghoff E, Morehead-Gee A, Hirozawa A, James C, Hammer H, Liu A, Gandhi M, Buchbinder S. Racial/ethnic and HIV risk category disparities in preexposure prophylaxis discontinuation among patients in publicly funded primary care clinics. Aids. 2019 Nov 15;33(14):2189-95.

Chambers LC, Morgan JL, Lowens MS, Robinson TS, Romano SS, Leipertz GL, Glick SN, Khosropour CM, Hughes JP, Golden MR, Fredricks DN. The Incidence Rate of Sexual Behaviors Among Cisgender Men who have Sex with Men Attending a Sexual Health Clinic. Archives of sexual behavior. 2023 Feb;52(2):751-9.

Laborde ND, Kinley PM, Spinelli M, Vittinghoff E, Whitacre R, Scott HM, Buchbinder SP. Understanding PrEP persistence: provider and patient perspectives. AIDS and Behavior. 2020 Sep;24(9):2509-19.

Comment: Page 3 of 33: "(2013/15/18)" is a confusing notation; "(2013, 2015, and 2018)" would be clearer.

Response: We also updated "(2013/15/18)" to "(2013, 2015, and 2018)".

Key Messages

Comment: Page 4 of 33: "What is already known on this topic: Men who have sex with men and transgender women are at high risk of HIV." Not every trans woman is at high risk of acquiring HIV. As we are in a global epi versus social epi/biobehavioral HIV science domain, this statement should

be qualified accordingly: “Men who have sex with men and transgender people are recognized by [PEPFAR/UNAIDS/etc.] as key populations at elevated risk of HIV acquisition.”

Response: This section has been updated based on Editor’s comment and now replaced with a strengths and limitations section.

Comment: Page 5-6 of 33: “HIV transmission in this group, like other KPs, is marked by involvement in high-risk sexual behaviours and practices including condomless sex, transactional sex, and injectable and non-injectable drug use [9 10]. These behaviours and practices intersect with other individual and societal level factors such as mental health and other health-related comorbidities, stigma, discrimination, and persecution [7 11]. Similarly, social challenges arising from structural barriers, such as limited chances for higher education or employment opportunities and lack of recognition of transgender persons’ gender by society, can also play a role in increasing the transmission of HIV within these populations, by limiting access to resources that promote health and protection [7 11 12].” This passage attempts to describe causal factors of disease (mal)distribution among trans people, acknowledging multilevel and interlocking drivers of sexual decision-making patterns to which we ascribe “risk” given the ongoing HIV epidemic. Presently, it lacks focus, portraying behaviors such as CAS and phenomena such as structural transphobia as occurring in parallel, rather than in an entangled, syndemic, manner. As this passage is meant to motivate the analysis, succinctly, I recommend grounding it in a cohesive theory of HIV disparity among trans people. The most comprehensive such framework is Reisner’s Situated Vulnerabilities and Resiliencies Framework (Reisner, 2025; https://doi.org/10.1016/S2352-3018(24)00299-6), a simple summary of which would accomplish more than this paragraph in its present state.

Response: We have revised the passage on pages 5–6 to better integrate the multilevel and interlocking drivers of HIV risk among transgender populations, grounding the discussion within Reisner’s *Situated Vulnerabilities and Resiliencies Framework* (Reisner, 2025). The revised text now emphasizes how individual behaviors, social challenges, and structural barriers interact to shape HIV vulnerability and reads as follows:

HIV transmission in this group is marked by entanglement of multilevel biopsychosocial factors [1], however, social, structural, and behavioural contexts play a profound role in shaping the HIV related vulnerabilities in these populations. At the individual level, behaviours such as engagement in high-risk sexual activities and substance use increases HIV acquisition risk [1-3]. These are often entangled with structural and societal level factors like stigma, discrimination, and lack of legal protection and recognition of the rights of transgender individuals which further compromises mental health of these individuals [1, 4-6]. Additionally, these multilevel factors cause inequalities in education, health access, employment. All of these factors sustain HIV vulnerabilities by creating a syndemic environment compounding HIV risk in such populations [1].

Comment: Page 5 of 33: The “KP” abbreviation is not used consistently, and the term itself occurs sporadically; it is not necessary to abbreviate. As well, precisely which standard-setting body (UNAIDS, PEPFAR, etc.) is defining “key populations” in the manner it is used in this paper should be stated explicitly.

Response: We no longer use the KP abbreviation and have mentioned that we are referring to the UNAIDS definition of KPs.

Page 6 of 33:

Comment: “Infection” is considered stigmatizing in contemporary HIV science. “Acquisition” should be used here (line 26).

Response: We have replaced the use of term “infection” with “HIV acquisition”

Comment: “IBBS” is never expanded throughout the manuscript. Given that this paper will likely be of interest to a transdisciplinary readership of health disparities and SGM health researchers, the Integrated Biological and Behavioral Surveillance approach should be very briefly defined.

Response: We have now updated this in abstract and methods section as Integrated biobehavioural Surveillance (IBBS) survey. In the methods we have added that “IBBS are used to monitor HIV epidemics by combining biological testing for HIV and other infections with surveys of behaviours and knowledge.”

Comment: References to the original IBBS reports presently lack URLs, and they are difficult to retrieve, lacking distinct identifiers. Please add the appropriate URLs.

Response: We have revised the references and included the URLs.

Comment: Page 7 of 33: “[...] biological men, who self-reported sexual contact (oral or anal) with another man in the
previous six months” is inaccurate here; aspects of medically affirmed trans women’s “biology” more closely align with that of cis women’s, and the total exclusion of trans men cannot be assumed given the IBBS methodology (as reported here). As birth-assigned sex is unlikely to have been ascertained in the midst of an RDS protocol, the more precise term would be “presumed assigned male at birth.” As the sample under consideration here is presumed trans women, “another man” should be replaced with “a man.”

Response: We have revised it to individuals who were presumed assigned male at birth, who self-reported sexual contact (oral or anal) with a man in the previous six months.

Comment: “Transgender status was characterised using responses to the question ‘Do you consider yourself as transgender’.” The presumption of a birth-assigned male sex and a “yes” endorsement to this item is sensitive enough, but not specific enough, to reliably encode every respondent who identifies as a woman, trans woman, or person on the trans-feminine spectrum. Indeed, it could as easily capture a nonbinary or questioning individual, or a trans man. While it is unsurprising that older population surveys in high-stigma contexts would fall short of contemporary SO/GI harmonization best practices, this limitation of the IBBS instrument, and the ambiguities in interpretation it introduces, must be acknowledged. Given that PEPFAR, UNAIDS, and the WHO recognize transgender and gender-diverse people, more broadly, as a population disproportionately burdened by HIV, the manuscript retains its value by more precisely characterizing the comparisons being reported here as transgender versus cisgender individuals who have sex with men, acknowledging that that supercategory definitionally includes TWSM in Ukraine, but may overrepresent the (presently unknowable) risk markers specific to TWSM in Ukraine.
I recommend providing the precise language of the “sex”-based inclusion criteria, and transgender status item, for each IBBS pooled here, in a text box or small table perhaps. Narratively, “transgender people” or “individuals” should be favored, with “transgender respondents, including trans women” used when discussing factors most salient (theoretically) to structural and interpersonal transmisogyny.

Response: The inclusion criteria for this survey were men who have sex with men, however, there was no specific question inquiring about the sex assigned at birth. We have still added a table describing the detailed MSM definition and the question assessing transgender status with possible options. We included this in the text: transgender status was assessed from a question related to transgender identity. Details of transgender item and definition of MSM, as used in the survey, are given in supplementary materials Table 1.

We also revised the narrative terminology to more precisely reflect the categories available in the dataset. Specifically, we now use “transgender people” when referring to the analytic group overall.

We have also acknowledged this as a limitation in the discussion: “Another challenge in this study was the use of term “men who have sex with men” for all study participants, which in the case of transgender women is unsuitable. We have used the term transgender women who have sex with men throughout. However, the sensitivity and specificity of the IBBS survey instrument to distinguish between nuanced transgender identities or gender-diverse categories has not been established in detail, likely resulting in some misclassification and inclusion of other gender-diverse individuals within the category of TGWSM.”

Comment: Page 8 of 33: “To account for potential confounding, we also adjusted our models for age (in years) and higher education attainment (completed undergraduate or higher degrees).” This particular control set should be justified theoretically. The precise operationalizations of these controls – e.g. whether age was entered as FEs, in strata, etc. – should be stated, either here or as a footnote to the appropriate tables.

Response: We added the justification in the text as follows: ” To account for potential confounding, we adjusted for age (as a continuous variable) and higher education attainment (completed undergraduate or higher degrees) by including these as additional covariates in our models. Age was selected since the vulnerability to HIV and engagement with HIV care could vary across the life course. Education attainment was selected to account for the potential influence of sociodemographic position on HIV outcomes and gender affirmation.”

Comment: “These measures were derived from a 15-item inner homophobia scale (Supplementary Tables 3 and 4).” This is another area where interpretation of these findings is muddied by the relative crudity of the IBBS instrumentation. Descriptively, the disparities reported in Supplementary Tables 3 and 4 are striking. But many (not all) of these stigma items are clearly contextualized “...because you have sex with men.” That stigmatization process is, therefore, predominantly in response to the respondents’ trans status and androphilic sexual behaviors. Qualitatively, there is likely a lot of case-by-case heterogeneity obscured by those estimates, including the stigmas particular to TWSM. This point should be covered in the Discussion.
At the same time, there are also powerful findings evident in the items (e.g. “afraid to seek medical help”; “afraid to be in public”) that lack the “because you have sex with men” qualifier. It is worth foregrounding these results, both because interpretation is a bit clearer, and the disparities equally as compelling.

Response: We added this point in discussion under the strengths and limitation section as: “Similarly, items within the stigma instrument, which have been validated among MSM in the US and Sub-Saharan Africa but not Eastern Europe, were specific to homosexuality-based stigma (androphilic sexual behaviours and male-assigned status). As such the significant levels of stigma and violence found among transgender participants within our study likely under-estimate the broader experiences of stigma among transgender participants. “

Results

Comment: Page 9 of 33: As BMJ Open is a generalist title, I recommend defining “chemsex” the first time the term is used (outside of the abstract).

Response: we have added in a definition in the first use outside of the abstract: chemsex [sex under the influence of recreational drugs]

Comment: a/ORs are notoriously difficult to interpret, and, for the effects the authors feel are most

implicative or compelling, should be accompanied by marginal effects. I recommend using Stata's post-estimation margins commands to provide the practical significance of these findings [e.g. "identifying as transgender was associated with greater odds of having a prior history of imprisonment (aOR=1.51, 95% CI: 1.00–2.31): a 0.18 uptick in probability of imprisonment relative to cisgender comparators"]. Whether average marginal effects, marginal effects at representative values, etc., are most appropriate, I leave to the authors. (Note that "0.18" is a toy value for illustrative purposes.)

Response: We estimated the average marginal effects. These are presented in supplementary material as table 4. In the results section, we added the following interpretation:

"The differences in predicted probabilities between transgender participants and cisgender MSM for experiencing each stigma and violence outcome are presented in supplementary table 4. The probabilities that transgender participants had experienced stigma from family and friends or general social stigma were 31% and 22% more (in absolute terms) than for cisgender MSM. Similarly, the probabilities of experiencing physical or sexual violence were 13-14% higher for transgender participants than those for CMSM."

Comment: "Transphobia, which is the societal discrimination of Persons whose gender identity and sexual practices do not correspond to societal standards." Transphobia involves the enforcement, via violence, the denial of opportunity, and the rescindment of bodily autonomy, of cishetero-patriarchal norms. It does not involve "sexual practices," focally, and should not be conflated with the specific stigmatization processes that afflict sexually minoritized communities – a point that is raised in Wesley King's review, cited here. (As well, there is a wealth of contemporary research on transphobia and trans-specific vectors of structural stigma; there is no reason to cite an article from 2006 to support this argument.) See my former point regarding grounding these analyses in a cohesive framework such as Reisner's, which will require raising these points (disambiguated from e.g. homophobia) in the Introduction.

Response: We have updated the discussion thoroughly.

Comment: In general, I recommend reserving the overview of structural/systemic drivers of HIV disparity, appropriately grounded, for the Introduction. The Discussion would more constructively focus on (actionable) implications, plus strengths and limitations, rather than "big picture" narratives.

Response: We have revised both introduction and the discussion sections. The revised discussion is now more focused on implications, comparison with other research, and strengths and limitations of the study.

Reviewer: 2
Dr. Alex Siu Wing Chan, The Hong Kong Polytechnic University

Comments to the Author:
The study aims to compare sexual risk behaviors, HIV care utilization, and experiences of stigma between transgender women who have sex with men (TGWSM) and cisgender men who have sex with men (CMSM) in Ukraine. After reviewing the manuscript, I believe the study presents a crucial topic and provides important insights into the disparities between these two populations. However, I have a few considerations and suggestions for improvement.

1. Introduction:
The introduction discusses the growing HIV epidemic in Eastern Europe and Central Asia, with a

particular focus on Ukraine. For my sense, while the context is well-established, I wonder if the introduction could benefit from a clearer explanation of how the findings specifically add to the existing literature. I believe the intersection between HIV risk behaviors and stigma could be explored further, particularly how these factors interact with socio-economic challenges. Expanding on these aspects would enhance the understanding of the gaps the study aims to address and potentially draw more attention to the importance of the research.

Response: we have updated our introduction, including a greater discussion of the HIV risk behaviours and stigma as well as socio-economic challenges:

"Evidence suggests that stigma and discrimination based on the gender identity of transgender persons can limit their engagement with HIV treatment and prevention services [6, 7], whilst there is inadequate access to HIV services that are tailored to the needs of transgender persons globally [8]. HIV transmission in this group is marked by entanglement of multilevel biopsychosocial factors [1], however, social, structural, and behavioural contexts play a profound role in shaping the HIV related vulnerabilities in these populations. At the individual level, behaviours such as engagement in high-risk sexual activities and substance use increases HIV acquisition risk [1-3]. These are often entangled with structural and societal level factors like stigma, discrimination, and lack of legal protection and recognition of the rights of transgender individuals which further compromises mental health of these individuals [1, 4-6]. Additionally, these multilevel factors cause inequalities in education, health access, employment. All of these factors sustain HIV vulnerabilities by creating a syndemic environment compounding HIV risk in such populations [1].

Research has also indicated lower levels of health-seeking by transgender persons [9]. While there is lack of comprehensive research exploring the underlying causes, some evidence attributed these low levels of health-seeking to stigma and discrimination faced at various levels: structural, societal, and individual [9]. Evidence has also indicated that internalisation of discriminatory attitudes and prejudice by transgender persons underly these low levels of healthcare utilisation [9]. This stigma can also lead to reduced knowledge about HIV prevention and condom usage [10]. A strong body of research has highlighted negative associations between structural and individual-level stigma and utilisation of HIV care, HIV testing, and PrEP use among diverse population groups [11-14]. Research also established links between stigma and reduced sexual health services utilisation due to and perceived fear of stigmatisation by healthcare professionals among Sexual and gender-minority populations[12, 14]. The intersection of homophobic and transphobic stigma with HIV status, sex work, ethnicity, and drug use are also believed to underly adverse HIV outcomes among populations [15, 16]."

2. Methods:

In the methods section, the study does a decent job of outlining the design, sampling strategy, and statistical techniques. However, I feel there needs to be further clarification regarding the use of respondent-driven sampling (RDS) and the absence of RDS weighting in the regression models. I am wondering if the rationale for excluding RDS weights could be better explained.

Response: we acknowledge the reviewer's point regarding the absence of RDS weighting in our regression models. While RDS weighting can be valuable for generating population-level prevalence estimates, there is an active debate about its appropriateness for regression modelling, where it may inflate variance and introduce instability. Following precedents in HIV epidemiology using IBBS data, we chose to report unweighted regression results, with the goal of minimizing model dependency on potentially fragile weights. We have clarified this rationale in the revised Methods section by adding the following text:

“We did not apply RDS weights in our analyses as its application in regression analysis remains contested due to their negative effects on variance and model stability as RDS weighting may result in increases in variance which can limit the validity of regression models [17, 18].”

Furthermore, the survey instrument’s validity and reliability are not sufficiently addressed. In my view, including a brief discussion on the potential limitations of the methodology would help to strengthen this section.

Response: We agree that the validity and reliability of the IBBS instrument merit explicit discussion. Although the IBBS is a widely implemented surveillance tool in Ukraine and internationally, it was not originally designed with sufficient sensitivity to capture sexual orientation and gender identity (SO/GI) at the granularity required for this study. We now note these limitations explicitly, emphasizing how they may shape the interpretation of our findings. In the Ukraine MSM-focused IBBS, stigma items measured experiences such as family/friend stigma and anticipated healthcare stigma. While these have been used in contexts like the U.S. and sub-Saharan Africa, they have not been formally validated in Ukraine and are unlikely to have been assessed for sensitivity/specificity with respect to different identity groups like transgender individuals.

Multiple IBBS implementations in countries like Nepal, Cambodia, Nigeria, and India have targeted MSM and transgender populations. Many use terms like “MSM/TG” in their sampling frames or questionnaires, but none of the available documentation appears to report psychometric validation of the SO/GI measures (nor sensitivity/specificity estimates)

Given these limitations of IBBS instruments, we added a paragraph on the validity and reliability of the survey instrument in the methods section as:

“…the sensitivity and specificity of the IBBS survey instrument to distinguish between nuanced transgender identities or gender-diverse categories has not been established in detail, likely resulting in some misclassification and inclusion of other gender-diverse individuals within the category of TGWSM. Similarly, items within the stigma instrument, which have been validated among MSM in the US and Sub-Saharan Africa but not Eastern Europe, were specific to homosexuality-based stigma (androphilic sexual behaviours and male-assigned status). As such the significant levels of stigma and violence found among transgender participants within our study likely under-estimate the broader experiences of stigma among transgender participants. “

Additionally, clearer details on how transgender participants were categorized in the analysis could improve transparency.

Response: We updated and added details of the qualifying question and criteria in supplementary materials in materials and expanded the Methods to describe more clearly how transgender participants were categorized in the analytic sample. Specifically, we added the following text to clarify this: transgender status was assessed from a question related to transgender identity. Details of transgender item and definition of MSM, as used in the survey, are given in supplementary materials Table 1.

We have also acknowledged that the available survey measures are imperfect proxies and may conflate heterogeneous groups. This limitation is now highlighted in the revised text, alongside implications for interpretation.

3. Results:

While the results section provides a comprehensive dataset comparing TGWSM and CMSM, I am considering that there could be more discussion on how these behavioral differences may influence

broader public health policies and interventions. The results would benefit from being summarized more clearly, particularly concerning HIV prevalence and care engagement. For my focus, I believe the presentation of tables and figures is essential, but clearer labeling and more direct references within the text would improve readability. Additionally, further elaboration on the clinical implications, particularly tailored interventions for TGWSM, would enhance this section.

How does the significant difference in mean age (27.67 for TGWSM vs. 28.83 for CMSM) impact the study's findings on sexual risk behaviors? I'm wondering what might explain the slightly higher proportion of TGWSM (3.26%) with a history of imprisonment compared to CMSM (2.05%). Why is there no significant difference in HIV prevalence (5.17% for TGWSM vs. 5.43% for CMSM)? I'm curious about the underlying reasons for the higher mean number of long-term sex partners in TGWSM (0.8) compared to CMSM (0.7). How do the findings on chemsex (7.77% in TGWSM vs. 4.7% in CMSM) influence HIV prevention strategies?

I also wonder what factors could explain the higher percentage of TGWSM receiving free condoms (58.65%) compared to CMSM (48.76%). Why was there no significant difference in the use of condoms for the last anal sex (74.16% for TGWSM vs. 75.80% for CMSM)? How do the results on commercial sex involvement (18.49% in TGWSM vs. 15.17% in CMSM) contribute to the overall risk assessment for HIV? The higher levels of stigma faced by TGWSM (41.60% reported stigma from family and friends vs. 30.59% in CMSM) make me think about their healthcare access—what implications does this have? Finally, how might the difference in fear of being in public (31.88% in TGWSM vs. 10.59% in CMSM) inform public health policies targeting these groups?

Response: We have made more clear and direct references to the tables and findings within the text. We have also included public health implications and tailored interventions for TGWSM in the discussion section. In relation to some of the specific queries raised we have edited our discussion to incorporate what (to maintain within word limits) we believe are some of the more important findings.

"Our findings demonstrated that CMSM and TGWSM both experienced high levels of stigma and violence – experiences which are thought to affect HIV risk behaviours and access to HIV prevention and care [19-23] and were associated with being HIV positive in our study. However, TGWSM were approximately 3 times more likely to experience stigma and violence than CMSM. Research has highlighted that transphobia is embedded in cisnormativity and heteropatriarchal societal norms [1, 6] and operates on multiple levels, including structural and interpersonal factors, which play a critical role in shaping the health-related vulnerabilities, access to care and prevention among transgender persons [1]. In our study, more than a third of transgender participants reported being fearful of seeking healthcare, while 20 percent avoided seeking healthcare due to this fear. Such high levels of anticipated healthcare stigma may not only significantly impact on engagement with HIV care among transgender individuals but their wider health and highlights the need for culturally sensitive care for transgender populations.

We also found that transgender participants were more likely to have a history of imprisonment than cisgender MSM, which may indicate disproportionate policies and structural barriers to transgender people's rights in communities. The intersection of transphobia, marginalisation, and structural limitations to transgender rights in parallel with economic vulnerabilities that arise from these societal barriers may explain these increased levels of incarceration. The same structural barriers can negatively influence opportunities in education, housing, employment, right to marry, and gender affirmation [24, 25]. These economic challenges that transgender persons face in society may explain the increased levels of commercial sex among TGWSM. There is a need for programs and policies that ensure social protection and equal opportunities for transgender and other sexual minority populations in education, employment, and access to healthcare.

Whilst we found greater levels of risk high-risk behaviours among transgender participants than cisgender participants, we did not demonstrate a statistically significant difference in HIV prevalence between the two population groups. This could be attributed to the low number (about 5%) of HIV positive individuals in each group limiting the statistical power. Indeed, previous studies have consistently demonstrated a strong correlation between risk and prevention behaviours and HIV positivity in MSM populations across various geographies [26-29]."

4. Discussion:
The discussion does an adequate job of interpreting the findings, but I feel it could offer a more detailed analysis of the social and structural factors contributing to the behaviors observed among TGWSM. After reviewing the manuscript, I think the discussion of stigma is important, but it could go further in addressing the role of institutionalized stigma, especially with regard to healthcare access. It would be helpful to make a more explicit connection between the statistical findings and their real-world implications for HIV prevention strategies. Additionally, comparing these results to similar studies in other regions could provide greater validation and offer a broader perspective on the findings.

Response: we have substantially revised the discussion section. Specifically, we now provide a more detailed analysis of the social and structural factors that shape HIV risk among TGWSM, including an expanded discussion of institutionalised stigma and its impact on healthcare access. We also strengthened the connection between our statistical findings and their practical implications for HIV prevention strategies.

5. Conclusion and Limitations:
The conclusion provides a brief summary of the study's findings but lacks a deeper reflection on the broader societal implications. In my opinion, it would be more impactful if it offered more practical recommendations for improving HIV care services specifically tailored to transgender populations. The limitations are mentioned, but for my sense, the potential for recall bias, social desirability bias, and underrepresentation of older individuals could be more explicitly acknowledged. I believe the study's external validity, particularly how generalizable the results are to other regions, needs more consideration. Expanding on how these findings could influence future research on transgender health would provide a valuable direction for subsequent studies.

Response: we have revised this section to include a more explicit discussion of the broader societal implications of our findings, particularly with regard to how institutional and social barriers shape HIV risk and care engagement for TGWSM. We have added practical recommendations for strengthening HIV care services that are tailored to the needs of transgender populations, such as improving provider competency, reducing healthcare stigma, and enhancing community-based outreach.

Additionally, we expanded the limitations section to more clearly acknowledge potential recall bias, social desirability bias, and the underrepresentation of older TGWSM in our sample as follows:

This study utilised data from three national integrated biobehavioural surveys of MSM in Ukraine, comprising data from over 18,000 respondents. The data included information about HIV testing and treatment, risk behaviours, stigma, and other negative experiences, as well as demographic information of the respondents, enabling us to explore behavioural practices, service utilisation patterns, and experiences of stigma faced by TGWSM and CMSM. The geographic representation of all regions of Ukraine and the large sample size increases the generalisability of the study findings. However, approximately 90% of the survey participants were below the age of 40, substantially higher than the general population demographics (45% adult males <40 years). The underrepresentation of older MSM and TGW in these surveys is a limitation of this data, as

commonly found in MSM surveys [30-32], and may limit the generalisability of the findings to older MSM and transgender populations.

Another limitation of this study is the transgender participants in the IBBS were involved through NGOs. Consequently, the findings pertaining to the engagement of transgender women with NGOs may not accurately reflect the broader patterns of NGO engagement in Ukraine. Moreover, there was a lack of questions on stigma and related negative experiences in earlier surveys, and so only data from 2018 was used to measure these outcomes. Responses to many questions about risk behaviours and experiences of stigma and violence may be subject to recall bias, as with other self-reported behavioural surveys. Particularly, experiences of stigma, violence, and victimisation may be discomforting to recall due to which participants may have avoided expressing these experiences. Additionally, social desirability or acquiescence bias due to stigma associated with homosexual partnerships, condomless intercourse, and chemsex, is likely as the participants may feel hesitant to report behaviours that they consider unacceptable to the society, On the other hand, behaviours such as HIV testing trends and PrEP use are likely to be over reported. However, it is unlikely that these biases would influence the responses of transgender and CMSM differently. Stigma towards sexual minorities is also widespread, which could have influenced MSM's ability to participate in the survey or express their true gender identity or sexual orientation in the survey.

Another challenge in this study was the use of term "men who have sex with men" for all study participants, which in the case of transgender women is unsuitable. We have used the term transgender women who have sex with men throughout. However, the sensitivity and specificity of the IBBS survey instrument to distinguish between nuanced transgender identities or gender-diverse categories has not been established in detail, likely resulting in some misclassification and inclusion of other gender-diverse individuals within the category of TGWSM. Similarly, items within the stigma instrument, which have been validated among MSM in the US and Sub-Saharan Africa but not Eastern Europe, were specific to homosexuality-based stigma (androphilic sexual behaviours and male-assigned status). As such the significant levels of stigma and violence found among transgender participants within our study likely under-estimate the broader experiences of stigma among transgender participants.

Finally, the findings of this study present an overview of the vulnerabilities of the MSM and TGW in Ukraine where stigma, discrimination, and violence against sexual and gender-minority is more prevalent than some other more progressive regions in Europe. As such our findings may not be generalisable to other regions in Central or Western Europe, or settings outside Europe, where sociocultural contexts and levels of stigma differ considerably. Whilst further research is needed to establish causal links between stigma and violence and HIV risk among MSM and transgender populations, it is important that this occurs across countries with differing sociocultural and legal and policy environments to better inform policy and programming for HIV prevention among MSM and TGW populations."

Finally, we outlined specific areas for future research on transgender health, "Whilst further research is needed to establish causal links between stigma and violence and HIV risk among MSM and transgender populations, it is important that this occurs across countries with differing sociocultural and legal and policy environments to better inform policy and programming for HIV prevention among MSM and TGW populations. "

VERSION 2 - REVIEW

**Reviewer**	**1**
**Name**	**Skeen, Simone**
**Affiliation**	**Tulane University**
**Date**	**06-Nov-2025**
**COI**	

The efforts to address both reviewers' feedback are comprehensive, made in good faith, and improve the paper. The new introduction and discussion passages, leaning into contemporary SGM and HIV work focused on structural/systemic determinants, is excellent. The new Table 4, in particular, is easily interpretable and paints a devastating picture of the disparities recorded by this work of research. I do, however, see further opportunities for improvement, particularly in the newly added materials at R1.

Abstract: The use of "men who have sex with men," "MSM," "TGWSM" and "CMSM" in the abstract is more, not less, confusing than in the original submission.

Strengths and Limitations: It is my understanding that RDS-enrolled cohorts are not nationally representative but rather represent a critical (and otherwise unobtainable) mass of otherwise "hard-to-reach" subpopulations. But I am not an RDS expert.

Overall, the muddiness in interpretation of the transgender identification item remains. One example: Table 5 includes "transgender persons" in the title and "Transgender Women" in the columns. I remain adamant in my stance that interpreting the IBBS "transgender" item as universally trans-feminine is reductive, and believe "transgender people who have sex with men" to be the (sole) accurate label to attach to that subgroup, parsimoniously, while acknowledging them as "transgender persons, including trans women, assigned male at birth" in the narrative sections. "Transgender" is not a gender identity; we don't know the gender identity of the trans people recruited in this survey. And while it is a safe assumption that most are trans women (again, not "MSM"; you can easily write "transgender people sampled as MSM"), it is likely some unknowable proportion of them identify as nonbinary – or something else. Setting aside questions of principle that inevitably inflect one's interpretation of this issue, as presently worded, particularly to the casual reader, the impression of two distinct subgroups is given in the R1.

This issue is acknowledged in the second-to-last paragraph of the Discussion. Though I believe a more comprehensive rewording is most apt, a quick solution would be to move the below language:

"However, the sensitivity and specificity of the IBBS survey instrument to distinguish between nuanced transgender identities or gender-diverse categories has not been

established in detail, likely resulting in some misclassification and inclusion of other gender-diverse individuals within the category of TGWSM."

into the Methods section – it is, at its heart, an issue of instrumentation, not interpretation.

I recommend provisional acceptance with these changes required prior to publication – though that decision is ultimately in the hands of the Editor.

**Reviewer**	**2**
**Name**	**Chan, Alex Siu Wing**
**Affiliation**	**The Hong Kong Polytechnic University**
**Date**	**12-Nov-2025**
**COI**	

I think the authors resolved most of the concerns.

VERSION 2 - AUTHOR RESPONSE

Comments to the Author:

Comment 1: Abstract: The use of "men who have sex with men," "MSM," "TGWSM" and "CMSM" in the abstract is more, not less, confusing than in the original submission.

Response: We have edited the abbreviations in the abstract to make the distinction between the population groups more explicit. Following UNAIDS and Korenkomp et al., we used TGWSM abbreviation (transgender women who have sex with men) terminology for the population group that identified with transgender status.

Korenromp EL, Sabin K, Stover J, Brown T, Johnson LF, Martin-Hughes R, Ten Brink D, Teng Y, Stevens O, Silhol R, Arias-Garcia S. New HIV infections among key populations and their partners in 2010 and 2022, by world region: a multisources estimation. JAIDS Journal of Acquired Immune Deficiency Syndromes. 2024 Jan 1;95(1S):e34-45.

Comment 2: Strengths and Limitations: It is my understanding that RDS-enrolled cohorts are not nationally representative but rather represent a critical (and otherwise unobtainable) mass of otherwise "hard-to-reach" subpopulations. But I am not an RDS expert.

Response: We have added the following text to highlight the limitations of RDS recruitment methods in the strengths and limitations section of the manuscript.

“As the participants in this study were recruited using the RDS methodology utilising chain referral sampling, the study findings cannot be generalised to the overall MSM populations across Ukraine and representative of the population groups reached through IBBS recruitment mechanism.”

Comment 3: Overall, the muddiness in interpretation of the transgender identification item remains. One example: Table 5 includes “transgender persons” in the title and “Transgender Women” in the columns. I remain adamant in my stance that interpreting the IBBS “transgender” item as universally trans-feminine is reductive, and believe “transgender people who have sex with men” to be the (sole) accurate label to attach to that subgroup, parsimoniously, while acknowledging them as “transgender persons, including trans women, assigned male at birth” in the narrative sections. “Transgender” is not a gender identity; we don’t know the gender identity of the trans people recruited in this survey. And while it is a safe assumption that most are trans women (again, not “MSM”; you can easily write “transgender people sampled as MSM”), it is likely some unknowable proportion of them identify as nonbinary – or something else. Setting aside questions of principle that inevitably inflect one’s interpretation of this issue, as presently worded, particularly to the casual reader, the impression of two distinct subgroups is given in the R1.

This issue is acknowledged in the second-to-last paragraph of the Discussion. Though I believe a more comprehensive rewording is most apt, a quick solution would be to move the below language:

“However, the sensitivity and specificity of the IBBS survey instrument to distinguish between nuanced transgender identities or gender-diverse categories has not been established in detail, likely resulting in some misclassification and inclusion of other gender-diverse individuals within the category of TGWSM.”

into the Methods section – it is, at its heart, an issue of instrumentation, not interpretation.

Response: We agree with the limitation of potential misclassification of gender minorities in MSM surveys, as highlighted by the Reviewer. We have now acknowledged this in more detail in the methods section as follows:

“Gender Identity and Sexual Behaviour Variable

Transgender status in the IBBS survey was assessed using a binary response question. The inclusion criteria for overall study population were if the sex assigned at birth was male and the individual had had sex with other men in past 6 months with another men. Accordingly, in this study, we refer to this subgroup as transgender women having sex with men, while some individuals in transgender group in IBBS may include inter alia individuals including transgender people and those who identify with nonbinary or other gender identities. “

This limitation was previously only mentioned in the strengths and limitations section. Additionally, we have updated the use of transgender identification terminology across the thesis to ensure consistency in the use of terminology representing transgender women in this study. Table 5 (supplementary materials) title now reads “TGWSM (transgender women)”.
